# Relationships between sport-specific tests and their validity in predicting the time-motion profile in international taekwondo matches

**DOI:** 10.1186/s13102-025-01371-4

**Published:** 2025-11-10

**Authors:** Gennaro Apollaro, Marco Panascì, Emerson Franchini, Gabriele Morganti, Piero Ruggeri, Coral Falcó, Emanuela Faelli

**Affiliations:** 1 Department of Neuroscience, Rehabilitation, Ophthalmology, Genetics and Maternal Child Health, University of Genoa, Genoa, Italy; 2 Centro Polifunzionale di Scienze Motorie, Università degli Studi di Genova, Genoa, Italy; 3 Department of Experimental Medicine, Section of Human Physiology, University of Genoa, Genoa, Italy; 4 Martial Arts and Combat Sports Research Group, Sport Department, School of Physical Education and Sport, University of São Paulo, São Paulo, Brazil; 5 Department of Human Sciences and Promotion of the Quality of Life, San Raffaele Roma Open University, Rome, Italy; 6 Department of Sport, Food and Natural Sciences, Western Norway University of Applied Sciences, Bergen, Norway

**Keywords:** Combat sports, Physical tests, Time-motion analysis, Competition, FSKT, Predictive criterion validity

## Abstract

**Background:**

The Frequency Speed of Kick Test (FSKT) and the Progressive Specific Taekwondo Test (PSTT) are among the most used sport-specific tests in taekwondo. This study aimed to: (i) investigate the relationship between sport-specific anaerobic (FSKT) and aerobic (PSTT) performance; (ii) evaluate these tests’ validity in predicting the time-motion profile in official matches.

**Methods:**

Sixteen national/international-level taekwondo athletes participated in this study. The FSKT and the PSTT were performed on consecutive days. Within the following 1–3 weeks, athletes competed in an international competition. Videos of each athlete’s first combat of the day were analyzed for time-motion analysis, including thirty-two performances (tested athletes and respective opponents), with a total of 2988 actions.

**Results:**

Multiple FSKT performance was correlated with aerobic power (Rho [ρ] = 0.730–0.759 [95% CI: 0.352–0.914], *p* ≤ 0.013) and capacity (ρ = -0.606 [95% CI: -0.852 – -0.141], *p* = 0.013) indicators of the PSTT. Time-motion indexes were correlated (ρ = 0.756–1.000 [95% CI: 0.402–1.000], *p* < 0.001) and the activity profile did not differ (*p* > 0.05) between tested athletes and their opponents. No significant relationship emerged between FSKT performances and time-motion indexes (ρ = -0.442–0.396 [95% CI: -0.776–0.753, *p* > 0.05), as well as between PSTT indicators and time-motion indexes (ρ = -0.462–0.462 [95% CI: -0.786–0.786], *p* > 0.05).

**Conclusions:**

The pattern of correlations emerged between sport-specific performances suggests that the dynamics of interaction between anaerobic and aerobic metabolism are crucial for maintaining the short and intermittent kicking actions. The rhythm of the first combat of the day, generated by the technical-tactical dynamics, justifies the inability to predict high- and low-intensity actions from the physical fitness variables. However, both the time-motion indexes and tests’ performance allow for the prescription of specific trainings. Time-motion indexes could be used to structure sport-specific high-intensity interval training (HIIT), while the ten seconds FSKT performance and the capacity and power indicators of the PSTT (i.e., kick frequency at the heart rate deflection point and maximal kick frequency, respectively) could be used to prescribe HIIT with short and long intervals.

**Supplementary Information:**

The online version contains supplementary material available at 10.1186/s13102-025-01371-4.

## Introduction

Physiological measurements, systematically collected during official matches in taekwondo athletes (blood lactate: 6.7–14.0 mmol∙L^− 1^; peak heart rate: 96–97% of maximal heart rate), have made it possible to formulate the assumption that the typical intermittent activity of combat involves high demands on both anaerobic and aerobic metabolism [1, 2]. In addition, estimates of relative contributions of energy systems (ATP-PCr: 19–33%; glycolytic: 3–9%; oxidative: 62–74%), conducted during the simulated matches, allowed identification of distinct roles and their interaction dynamics, aimed at maintaining the short and repeated actions of kicking and punching [3–6]. Overall, these physiological data highlight the importance of implementing testing protocols to monitor both anaerobic and aerobic components, as both are essential for sustaining the typical activity performed during the match.

Over the past decade, several sport-specific tests have been developed and validated. These tests allow the assessment and monitoring of the anaerobic and aerobic components of the taekwondo athlete from an improved ecological perspective [7, 8]. The Frequency Speed of Kick Test (FSKT) and Progressive Specific Taekwondo Test (PSTT) are among the most used sport-specific tests in taekwondo [7, 8]. The FSKT is divided into two versions to assess anaerobic power (FSKT_10s_) and capacity (FSKT_mult_) in short and intermittent modes, respectively [9]. The PSTT allows the determination of both aerobic capacity and power indicators in continuous and progressive maximal mode [10]. Albuquerque et al. [11] examined the relationship between FSKT_mult_ performance and some aerobic power indicators of the PSTT in high-level Brazilian taekwondo athletes. A positive and significant relationship (*r* = 0.83) emerged between the total number of kicks in the FSKT_mult_ and the time to exhaustion of the PSTT. The authors hypothesized that high aerobic power would induce faster resynthesis of PCr between high-intensity efforts, optimizing FSKT_mult_ performance [11]. It is important to highlight that the FSKT_10s_ performance and aerobic capacity indicators of the PSTT were not included in their analyses, as well as the study was a secondary analysis derived from investigations with different procedures. Moreover, to date no other studies have investigated the relationship between the two sport-specific tests limiting their understanding [7].

The FSKT and PSTT have followed a relevant path of development and validation [9, 10]. Specifically, the FSKT has logic translational validity, discriminant construct validity, sensitivity, and test-retest and intra-/inter-rater reliability [8]. The PSTT has concurrent criterion validity and test-retest reliability [7]. Recent reviews on sport-specific assessment in taekwondo showed that predictive criterion validity has never been investigated among anaerobic and aerobic tests [7, 8]. Previously, Chaabene et al. [12] found that this aspect of validity has been surprisingly neglected for sport-specific tests in Olympic combat sports, despite its fundamental importance in practice for coaches and strength and conditioning professionals. In order for a sport-specific test to be an effective measurement tool, it must be valid, reliable, and sensitive [12]. In this sense, recent studies in other combat sports such as karate [13] and kickboxing [14], or in other sports contexts [15], have established various aspects of validity, reliability, and sensitivity of sport-specific tests in parallel, supporting their widespread use in practice. According to Apollaro et al. [8], the absence of studies that have assessed predictive criterion validity among taekwondo sport-specific tests, and more generally the lack of studies in Olympic combat sports, could indicate some difficulty in structuring experimental designs involving official matches. In this context, the use of simulated matches, with the necessary adjustments to standardize the experimental protocol and ensure the safety of athletes and equipment, is an alternative approach to studying this validity aspect [16]. However, it is crucial to highlight that this study strategy limits the applicability of the knowledge gained to the official competitive environment. Indeed, official taekwondo matches are held within regulated competitions (with referees and implications for rankings and medals), which can consequently induce distinct physiological responses, greater commitment to techniques, highly specific combat strategies, and higher levels of motivation than the simulated matches. In addition, the official match is influenced by typical situations on competition day that involve resource management, as athletes may face 4–5 combats on the same day [7, 8].

The time-motion analysis is a methodology used in combat sports to quantify the time and frequency spent in the different kinds of actions performed [2]. In this regard, estimates of relative contributions of energy systems in taekwondo simulated matches, mentioned above, were generated by an attack/skipping ratio between ~ 1:3 and 1:7, with attack times lasting between 0.7 and 1.5 s [3–6]. While the estimates provide insights about the role of each energy metabolism, the specific rhythm generated by the technical-tactical dynamics between the two interacting opponents provides practical information about how to structure general and specific training programs [17, 18]. Moreover, the time structure of international taekwondo competition seems to be unmodulated by the outcome of the match, since in the high-level competitions the differences in performance ability between athletes might be minimal. At same time, the systematic rule changes, aimed at enhancing combat activity, might equalize the activity profile of interacting athletes [19, 20]. It is important to highlight that time-motion analysis is non-invasive and applicable regardless of the simulated or official nature of the match [3, 5, 19, 20]. Thus, it may represent a useful approach to investigate the predictive relationships between sport-specific anaerobic and aerobic performance and the active (attack and skipping) and passive (pauses) phases of the official taekwondo match. This approach would improve the experimental external validity of the study design for investigating predictive criterion validity, optimizing the applicability of the knowledge obtained to the official competitive environment.

Therefore, this study aimed to: (i) investigate the relationship between taekwondo sport-specific anaerobic (FSKT_10s_ and FSKT_mult_) and aerobic (PSTT) performance; (ii) evaluate the validity of the above sport-specific tests in predicting the time-motion profile in international taekwondo matches. It was hypothesized: (a) the presence of significant relationships between sport-specific anaerobic and aerobic performance; (b) the absence of significant relationships between sport-specific performance and time-motion indexes of the match. In this sense, the specific rhythm generated between the two interacting opponents could justify the absence of relationships between physical fitness variables and the time and frequency spent in the different kinds of actions performed.

## Methods

### Study design

In this cross-sectional observational study, athletes conducted three distinct experimental sessions. The first two sessions were conducted on consecutive days while the third session in the following 1–3 weeks. On the first day, anthropometric and body composition measurements, and sport-specific anaerobic tests (FSKT_10s_ and FSKT_mult_) were performed. On the second day, after 24 h, athletes carried out the sport-specific aerobic test (PSTT). On the last day, athletes participated in an official international competition recognized by World Taekwondo (WT).

### Participants

Required sample size was derived a priori using G*Power software (*v. 3.1.9.7; Heinrich Heine University in Düsseldorf*,* Germany*) for a bivariate normal model (two-tailed) using the following parameters: *r* = 0.70; α = 0.05; β = 0.85; sample size required = 15. The expected *r* value was based on the strong correlation (*r* = 0.83) between the total number of kicks in the FSKT_mult_ and the time to exhaustion of the PSTT, previously reported [11]. Sixteen national/international-level taekwondo black belt athletes (8 males [6 juniors and 2 seniors], 8 females [4 juniors and 4 seniors]; weight category: 2 = flyweight; 1 = bantamweight; 3 = featherweight; 4 = lightweight; 3 = welterweight; 2 = light middleweight; 1 = middleweight), from the same club, took part in this study (Table 1). Inclusion criteria were: having more than three years of experience in taekwondo competitions; being training at least five times a week; not having suffered muscle and joint injuries in the past 6 months; not having taken drugs, medications or dietary supplements; not having engaged in any acute rapid weight loss strategies during the experimental period. In this regard, no significant difference was observed between the body mass measured during the first experimental session and that measured at the official pre-competition weigh-in (58.9 ± 6.9 kg; t_15_ = 0.286, *p* = 0.778, Cohen’s *d*: 0.072 [95% CI: -0.420–0.561]). All the athletes were informed about the experimental procedure. They provided written informed consent (or their parents for athletes under the age of 18) before participating in this study, which was approved by the Local Ethics Committee (University of Genoa—approval no: 2024/44) and performed in accordance with the Helsinki Declaration [21].

**Table 1:** Characteristics of athletes participating in the study. Values are presented as mean (standard deviation) [minimum–maximum]

Characteristics	Total (*n* = 16)		
	Mean	SD	Min–Max
Age (year)	17.4	2.5	15–23
Experience (year)	12.0	2.3	8–17
Training/week (hour)	12	1	11–13
Body height (cm)	172.0	4.3	163–180
Body mass (kg)	59.0	6.5	49.7–72.9
Body fat (%)	15.0	8.9	4.9–31.6

### Procedures

**Fig. 1:**
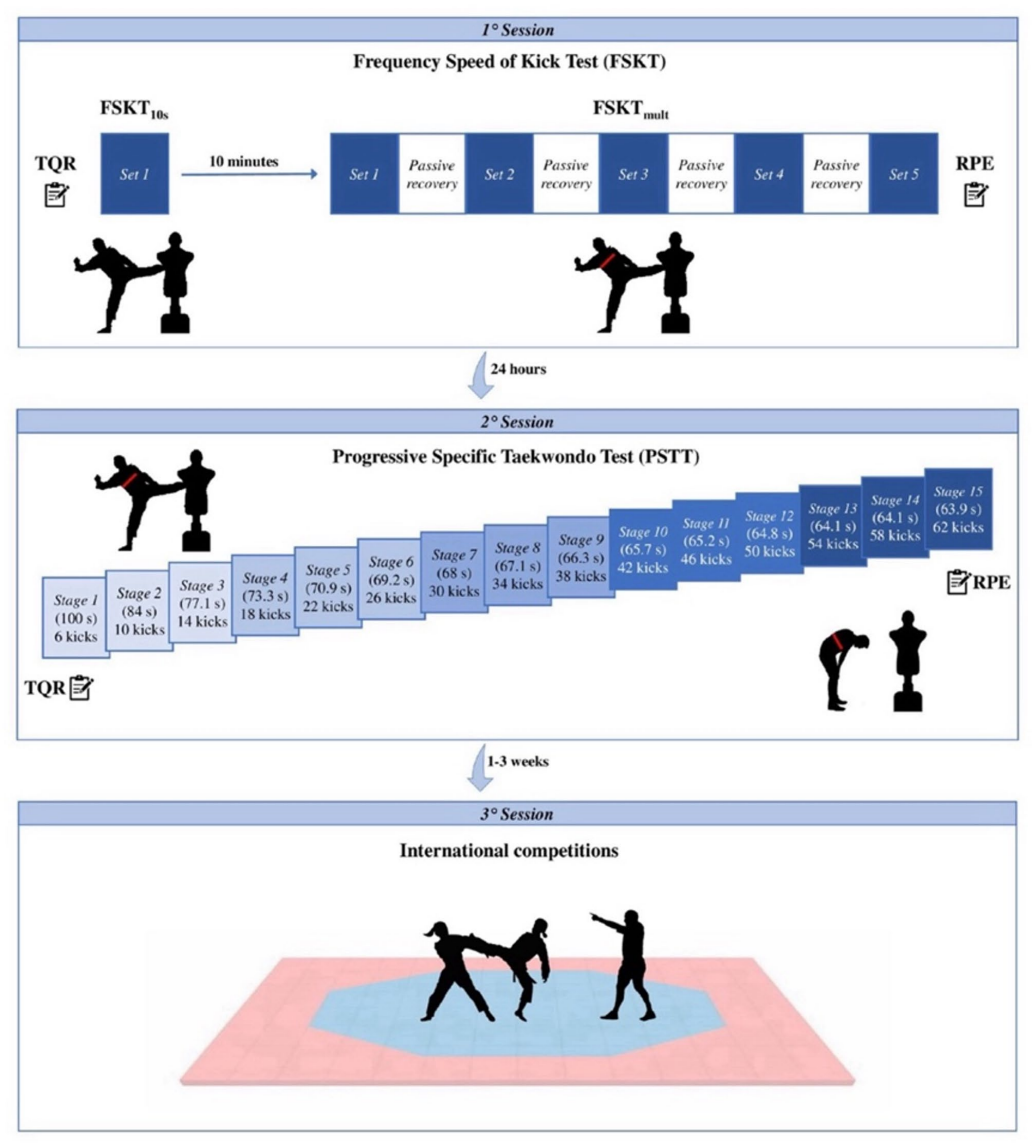
Schematic representation of the study design. Notes: TQR: total quality of recovery scale; FSKT_10s_: 10 s Frequency Speed of Kick Test; FSKT_mult_: Multiple Frequency Speed of Kick Test; RPE: rating of perceived exertion scale; s: second

In the week preceding the testing sessions, athletes completed two familiarization sessions with all the procedures to minimize the learning effect [12]. The data collection was conducted in the mid-phase of the competitive season, when athletes were engaged in regular competition and training loads were stable. The first two sessions were held at the athletes’ sports center, by main researcher (a taekwondo coach, ≥ 20 years of taekwondo experience and black belt), at the same time of day (10:00–12:00 a.m.), under similar temperature and humidity conditions (24–27 °C and 46–50%, respectively) to avoid any influence of the circadian rhythms. In the 24 h before these two sessions, athletes were asked to avoid consumption of caffeine and alcohol, any strenuous physical activity, and not to consume food for 2 h before the tests. Body height and composition were measured with a stadiometer (*Seca Model 217; SECA GmbH & Co. KG.*,* Hamburg*,* Germany*) and a bioelectrical impedance scale (*Tanita BC-420 MA; Tanita Corp.*,* Tokyo*,* Japan*), with 0.1 cm and 0.1 kg resolution, respectively. The first session was preceded by a standardized warm-up routine, for a total of 15 min [9]. After 5 min of passive recovery, athletes performed the FSKT_10s_ and, after a passive recovery of 10 min, the FSKT_mult_ following the test order previously reported [8]. The second session began with a standardized warm-up routine, for a total of 5 min [11, 22]. After 5 min of passive recovery, athletes performed the PSTT. The recovery state was recorded 15 min before these sessions using the TQR scale [23]. Immediately at the end of FSKT_mult_ and PSTT [24, 25], the perceived exertion was evaluated using the Borg 6–20 RPE scale [26]. HR was continuously measured, beat-by-beat, during the FSKT_mult_ and the PSTT using a heart rate monitor strap (*Polar H10; Polar Electro Oy*,* Kempele*,* Finland*). In the third session, each athlete participated in an official international competition (i.e., G-1/E-1 or E-2 ranking), in his/her respective age and weight category, following the WT competitive rules [27]. Since each athlete can make a variable number of matches based on the athletes in his/her weight category and number of matches won, only the first match of each athlete was considered. Thus, six videos of combats from the *15th Slovenia Open G-1/E-1* (Ljubljana, Slovenia – 24/25 February 2024), eight from the *11th Top Tirana Open E-2* (Tirana, Albania – 09/10 March 2024), and two from the *11th Ramus Sofia Open E-2* (Sofia, Bulgaria – 16/17 March 2024) were obtained for the time-motion analysis. In these sixteen matches, each athlete was studied individually and consequently thirty-two performances (the tested athlete and the respective opponent), with a total of 2988 actions, were analyzed. The experimental procedures are detailed in Fig. 1.

#### 10s frequency speed of kick test

The FSKT_10s_ lasts for 10 s (Fig. 1). The test execution criteria have been previously described [8, 9]. The performance was determined by the total number of kicks applied (FSKT_10s_).

#### Multiple frequency speed of kick test

The FSKT_mult_ consists of five 10 s sets with a 10 s passive recovery between sets (Fig. 1). The test execution criteria have been previously described [8, 9]. The performance was determined by the total number of kicks (FSKT_TOTAL_) and the kick decrement index (KDI) calculated using the Eq. (1) (see Additional file 1).

During the FSKT_mult_, a *Polar H10* monitor strap was used to record the following variables: mean heart rate (HR_MEAN_) which is the mean heart rate recorded during the test, peak heart rate (HR_PEAK_) which is the highest heart rate reached during the test, and HR_PEAK_ expressed as percentages of the actual maximal HR in the PSTT (%HR_MAX_).

Both tests were recorded, and the videos were subsequently analyzed using *Kinovea* software (*v. 0.9.5; Joan Charmant and Contributors*,* Bordeaux*,* France*) to manually count the valid kicks in frame-by-frame mode with an accuracy of 0.03 s. First, the count of a kick started when the athlete moved the attack foot and finished when he/she touched the bag. Kicks considered were those that hit the target during the 10 s. If the athlete started the kick before completing 10 s but reached the target only after 10 s, the kick was not considered. Second, valid kicks were those performed with appropriate technique and power [8, 9]. The main researcher quantified the valid kicks twice, by separating each observation by a 7-day interval, to verify the intra-rater reliability. In parallel, a second researcher (a taekwondo coach, ≥ 30 years of taekwondo experience and black belt) quantified the valid kicks to establish the inter-rater reliability. In agreement with the literature [8], FSKT_10s_ and FSKT_total_ showed an excellent relative (intraclass correlation coefficient [ICC] >0.9) and absolute (coefficient of variation [CV%] < 5) intra-/inter-rater reliability. Furthermore, considering recent advances in the sport-specific tests validation, in combat sports [12] and other sports contexts [28, 29], sensitivity and minimal detectable change in intra-/inter-rater analysis were analyzed. FSKT_10s_ and FSKT_total_ showed a good sensitivity, given that standard error of measurement (SEM) values were smaller than the smallest worthwhile change (SWC) ones. In addition, minimal detectable change at 95% confidence interval (MDC_95_) for these performances were small. On the other hand, KDI showed acceptable absolute intra-/inter-rater reliability (CV% <15) and marginal sensitivity (SEM >SWC) (Table 2).

**Table 2:** Relative and absolute intra-/inter-rater reliability and minimal detectable change at 95% confidence interval of sport-specific anaerobic (FSKT_10s_ and FSKT_mult_) performance (*n* = 16)

	Intra-rater reliability					Inter-rater reliability				
	Evaluator 1					Evaluator 1 and Evaluator 2				
FSKT	ICC [95% CI]	CV%	SEM	SWC	MDC_95_	ICC [95% CI]	CV%	SEM	SWC	MDC_95_
FSKT_10s_ (n° kicks)	0.992 [0.978–0.997]*	0.25	0.14	0.28	0.39	0.992 [0.978–0.997]*	0.25	0.14	0.28	0.39
FSKT_1_ (n° kicks)	0.985 [0.959–0.995]*	0.49	0.21	0.29	0.57	0.985 [0.959–0.995]*	0.49	0.21	0.29	0.57
FSKT_2_ (n° kicks)	0.979 [0.941–0.993]*	0.47	0.17	0.24	0.47	0.956 [0.850–0.985]*	0.94	0.24	0.24	0.65
FSKT_3_ (n° kicks)	0.984 [0.956–0.994]*	0.24	0.14	0.20	0.38	0.954 [0.861–0.984]*	0.76	0.22	0.20	0.61
FSKT_4_ (n° kicks)	0.987 [0.963–0.995]*	0.27	0.11	0.22	0.30	0.987 [0.963–0.995]*	0.27	0.11	0.22	0.30
FSKT_5_ (n° kicks)	0.921 [0.772–0.972]*	1.34	0.33	0.24	0.91	0.890 [0.694–0.961]*	1.88	0.37	0.23	1.03
FSKT_TOTAL_ (n° kicks)	0.988 [0.967–0.996]*	0.44	0.53	1.07	1.48	0.988 [0.966–0.996]*	0.45	0.53	1.06	1.46
KDI (%)	0.907 [0.742–0.967]*	10.70	0.83	0.55	2.29	0.886 [0.668–0.960]*	10.68	0.91	0.55	2.53

Notes: FSKT: Frequency Speed of Kick Test; FSKT_10s_: total number of kicks in FSKT_10s_; FSKT_1_: FSKT_mult_ set 1; FSKT_2_: FSKT_mult_ set 2; FSKT_3_: FSKT_mult_ set 3; FSKT_4_: FSKT_mult_ set 4; FSKT_5_: FSKT_mult_ set 5; FSKT_TOTAL_: total number of kicks in the 5 sets of FSKT_mult_; KDI: kick decrement index; ICC: intraclass correlation coefficient; CV: coefficient of variation; SEM: standard error of measurement; SWC: smallest worthwhile change; MDC_95_: minimal detectable change at 95% confidence interval. * = *p* < 0.001

#### Progressive specific taekwondo test

The first stage of the PSTT began with 6 bandal-chagi, alternating legs, and then progressively increasing 4 bandal-chagi on each new stage, as it is characterized by continuous and progressive maximal mode (Fig. 1). The test execution criteria have been previously described [7, 10]. Sound signals were transmitted from a smartphone using the *ITStriker* app (*ETS4ME*,* São José*,* SC*,* Brazil*). The following criteria was used to establish the end: (1) the athlete fails to track the KF (determined by beep); (2) the athlete performs the kicks without maintaining the technical and/or power standard effectively; or (3) the athlete stops the test (volitional exhaustion). A tolerance not exceeding 2 consecutive technical failures is allowed [7, 10].

During the PSTT, the *ITStriker* app and a *Polar H10* monitor strap paired with the app were used to record the following indicators: (1) maximal heart rate (HR_MAX_) which is the heart rate recorded at the end of the test; (2) maximal kick frequency (KF_MAX_) which is the highest frequency of kicks reached in the last stage of the test; (3) heart rate deflection point (HR_DP_); (4) kick frequency at the HR_DP_ (KF_DP_); (5) total number of kicks (K_TOTAL_); (6) time to exhaustion.

Similarly to previous studies [10, 30, 31], the HR_DP_ was identified using the D_MAX_ method [32, 33]. The D_MAX_ method does not present significant differences from maximal lactate steady state velocity and there was a high agreement between them (through Bland and Altman analysis) [34]. The HR curve was adjusted versus the KF obtained at each stage of the PSTT by a polynomial function of third order. Then, the first and last points of the curve were connected by a straight line, and the most distant point of the curve to the line was considered as the HR_DP_. Only values equal or greater than 140 b.min^− 1^ were used [34, 35].

#### Time-motion analysis

All matches were recorded by the *reStrike IVR* channel (within the open source website: https://www.youtube.com*)*, which is the Instant Video Replay service provider for the taekwondo. This ensured the time-motion analysis accuracy (i.e., excellent video quality with a fixed, complete shot of the competition area from approximately 1 m away). The videos were analyzed using *Kinovea* software in frame-by-frame mode with an accuracy of 0.03 s. The attack time (AT), number of AT (AN), skipping time (ST) and pause time (PT) were registered for each athlete (Table 3) [19, 20]. The following indexes were calculated from these phases: (1) average AT for each match; (2) average ST for each match; (3) average PT for each match; (4) average AT/ST ratio for each match; (5) average (AT + ST)/PT ratio for each match; (6) sum of AT for each match; (7) sum of ST for each match; (8) sum of PT for each match [19, 20]. Since each match had its own duration based on age category, number of rounds held, and method of victory, according to the WT competitive rules [27], the sum of AT and the sum of ST were relativized according to the total match time, and the following indexes were also calculated: (9) percentage AT; (10) percentage ST; 11) average ATsum/STsum ratio for each match; 12) average (ATsum + STsum)/PTsum ratio for each match. On the contrary, the number of attacks was not considered in this study as its relativization would not have added any information compared to the other indexes and would have made its interpretation difficult. In agreement with the literature [3–6, 19, 20, 36], time-motion indexes showed an excellent relative (ICC >0.9) and absolute (CV%<5) intra-/inter-rater reliability. In parallel, time-motion indexes showed a good sensitivity, given that SEM values were smaller than SWC ones. In addition, MDC_95_ for these indexes were small. On the other hand, AT_AVG_/ST_AVG_ ratio showed very good absolute inter-rater reliability (CV%<10) and satisfactory sensitivity (SEM = SWC) (Table 4).

**Table 3:** Time-motion parameters of the Taekwondo match

Action	Definition	Description	Start action	End action
**Active phase**				
Attack Time (AT)	The attack time is the total time during which the athlete attacks or tried to attack the opponent.	The attack time is considered as an active phase that includes technical exchanges (e.g., kicking, punching, and blocking techniques) between opponents and tactical movements (e.g., feints, changing stances and directions, steps etc.) preceding the attack with the aim of confusing or surprising the opponent, to score.	The attack time begins when, from the fighting stance, the foot leaves the floor to deliver the first kick (or support an offensive punch action) in the combat exchange between opponents. In case a tactical movement precedes the first offensive action (i.e., kicking or punching), the beginning of the attack time is considered when both feet move or leave the floor to initiate the tactical movement.	The attack time is considered ended when: (1) the foot that delivers the last kick of the action touches the floor; (2) the punching or blocking limb is retracted; (3) a “knock down” count of the referee as a consequence of a staggering blow hit; or (4) the referee uses the stop hand signal.
**Active phase**				
Skipping Time (ST)	The skipping time is defined as the total time in which there is no attempt to attack.	The skipping time is considered as an active phase that includes strategy planning, observation and physical preparation for the attack (e.g., judging safety distances, changing stances and directions, planning feints, and steps).	The skipping time begins either after the referee’s hand signal or upon termination of the fighting.	The skipping time ends either upon the referee’s hand signal or when the successive fighting time starts.
**Passive phase**				
Pause Time (PT)	The pause time is characterized by time-outs determined by the referees.	The pause time is a passive phase for: (1) solving technical problems; (2) giving warnings; (3) administering first aid to an injured athlete (one-minute); (4) replaying videos following an objection to a judgment.	The pause time begins either after the referee’s hand signal.	The pause time ends either upon the referee’s hand signal.

**Table 4:** Relative and absolute intra-/inter-rater reliability and minimal detectable change at 95% confidence interval of time-motion analysis (*n* = 32)

	Intra-rater reliability					Inter-rater reliability				
	Evaluator 1					Evaluator 1 and Evaluator 2				
Time-Motion Indexes	ICC [95% CI]	CV%	SEM	SWC	MDC_95_	ICC [95% CI]	CV%	SEM	SWC	MDC_95_
AT_AVG_ (s)	0.992 [0.983–0.996]*	1.25	0.06	0.13	0.18	0.955 [0.908–0.978]*	4.59	0.15	0.14	0.41
ST_AVG_ (s)	0.999 [0.998–0.999]*	0.81	0.03	0.17	0.07	0.987 [0.974–0.994]*	3.10	0.09	0.18	0.24
PT_AVG_ (s)	1.000 [1.000–1.000]*	0.09	0.00	2.12	0.00	1.000 [0.999–1.000]*	0.98	0.00	2.13	0.00
AT_AVG_/ST_AVG_ ratio	0.997 [0.994–0.999]*	1.81	0.02	0.09	0.06	0.964 [0.926–0.982]*	5.73	0.09	0.09	0.25
(AT_AVG_ + ST_AVG_)/PT_AVG_ ratio	1.000 [0.999–1.000]*	0.91	0.00	0.14	0.00	0.996 [0.991–0.998]*	2.69	0.04	0.14	0.11
AT_SUM_ (s)	0.999 [0.998–1.000]*	1.04	0.99	6.58	2.73	0.999 [0.998–0.999]*	1.17	0.98	6.56	2.72
ST_SUM_ (s)	1.000 [0.999–1.000]*	1.03	0.00	9.52	0.00	0.999 [0.999–1.000]*	1.15	1.43	9.52	3.95
PT_SUM_ (s)	1.000 [1.000–1.000]*	0.05	0.00	39.49	0.00	1.000 [1.000–1.000]*	0.18	0.00	39.49	0.00
AT_SUM_ (%)	0.998 [0.996–0.999]*	1.04	0.48	2.42	1.34	0.997 [0.995–0.999]*	1.17	0.60	2.42	1.67
ST_SUM_ (%)	0.998 [0.996–0.999]*	0.99	0.48	2.42	1.34	0.997 [0.995–0.999]*	1.10	0.60	2.42	1.67
AT_SUM_/ST_SUM_ ratio	0.997 [0.993–0.998]*	2.03	0.03	0.11	0.07	0.994 [0.988–0.997]*	2.27	0.04	0.11	0.12
(AT_SUM_ + ST_SUM_)/PT_SUM_ ratio	1.000 [1.000–1.000]*	0.05	0.00	0.30	0.00	1.000 [1.000–1.000]*	0.18	0.00	0.30	0.00

Notes: AT_AVG_: average attack time; ST_AVG_: average skipping time; PT_AVG_: average pause time; AT_SUM_: sum of attack time; ST_SUM_: sum of skipping time; PT_SUM_: sum of pause time; ICC: intraclass correlation coefficient; CV: coefficient of variation; SEM: standard error of measurement; SWC: smallest worthwhile change; MDC_95_: minimal detectable change at 95% confidence interval. * = *p* < 0.001

### Statistical analysis

Data analyses were performed using *Jamovi* software (*v. 2.3.28*; *The Jamovi Project*,* Australia*). Relative inter-/intra-rater reliability of the FSKT and the time-motion analysis was computed using an average measures two-way random ICC with absolute agreement and 95% confidence intervals. The ICC values were interpreted as follows: <0.5: poor; 0.5–0.75: moderate; 0.75–0.9: good; >0.9: excellent [37]. While, absolute inter-/intra-rater reliability was expressed in terms of SEM and CV. The CV values were interpreted as follows: <5%: excellent; <10%: very good; <15%: acceptable; >15%: poor [38]. The sensitivity of the FSKT and the time-motion analysis was assessed by comparing the smallest worthwhile change (SWC) and SEM, using the thresholds proposed by Liow and Hopkins [39]. MDC_95_ was also calculated for FSKT and the time-motion analysis. The Kolmogorov-Smirnov test revealed that all the considered variables significantly deviated from a normal distribution. Given the violation of the normality assumption, data are presented as median, interquartile range [minimum–maximum], which better describe the central tendency and variability of non-normally distributed data. Spearman’s Rho (ρ) correlation coefficient, with 95% confidence intervals, was used to examine relationships between sport-specific anaerobic (FSKT_10s_ and FSKT_mult_) and aerobic (PSTT) performance. The magnitude of correlations was assessed using the criteria proposed by Hopkins et al. [38]: <0.1; trivial; 0.1–0.3: low; 0.3–0.5: moderate; 0.5–0.7: large; 0.7–0.9: very large; >0.9: nearly perfect; =1: perfect. To control for the proportion of statistically significant results that might be erroneously apportioned within the correlation analyses, leading to an increased chance of making a type 1 error, the Benjamini-Hochberg procedure (False Discovery Rate) was applied [40]. In addition, Spearman’s ρ correlation coefficient was used to investigate the relationships between sport-specific performance (FSKT_10s_, FSKT_mult_, and PSTT) and time-motion indexes, as well as these latter between the tested athletes and their opponents during international matches. The Mann-Whitney *U* test was used to determine whether time-motion indexes were different between tested athletes and their opponents. Effect size *r* was calculated by the formula: *r* = Z/√N; values of 0.1, 0.3 and 0.5 were considered to have small, medium and large effects, respectively [41]. The statistical significance was accepted when *p* < 0.05.

## Results

Table 5 shows sport-specific anaerobic (FSKT_10s_ and FSKT_mult_) and aerobic (PSTT) performance.

**Table 5:** Sport-specific anaerobic (FSKT_10s_ and FSKT_mult_) and aerobic (PSTT) performance in tested athletes. Values are presented as median (1st quartile–3rd quartile) [minimum–maximum]

Variables	Tested athletes (*n* = 16)		
Sport-Specific Anaerobic Performance	Median	1st Q–3rd Q	Min–Max
TQR (a.u.)	17	16–18	14–20
FSKT_10s_ (n° kicks)	19	18–19	17–23
FSKT_TOTAL_ (n° kicks)	86	83–90	82–102
KDI (%)	7	5–9	2–13
HR_MEAN_ (b.min^− 1^)	176	171–180	161–194
HR_PEAK_ (b.min^− 1^)	184	181–191	171–198
%HR_MAX_	96	94–96	91–98
RPE (a.u.)	15	15–16	14–20
Sport-Specific Aerobic Performance			
TQR (a.u.)	18	16–18	14–20
HR_MAX_ (b.min^− 1^)	190	186–195	178–206
KF_MAX_ (k.min^− 1^)	43	37–46	34–51
HR_DP_ (b.min^− 1^)	168	163–176	151–198
HR_DP_ (%HR_MAX_)	89	85–93	82–96
KF_DP_ (k.min^− 1^)	17	16–23	15–26
KF_DP_ (%KF_MAX_)	43	39–50	35–63
K_TOTAL_ (n° kicks)	300	218–344	186–409
Time to exhaustion (s)	825	695–880	655–958
RPE (a.u.)	17	16–20	15–20

Notes: TQR: total quality of recovery scale; FSKT_10s_: total number of kicks in the 10 s Frequency Speed of Kick Test; FSKT_TOTAL_: total number of kicks in the 5 sets of the Multiple Frequency Speed of Kick Test; KDI: kick decrement index; HR_MEAN_: mean heart rate; HR_PEAK_: peak heart rate; %HR_MAX_: percentages of the athlete’s HR in the PSTT; RPE: rating of perceived exertion (Borg 6–20 scale) scale; HR_MAX_: maximal heart rate; KF_MAX_: maximal kick frequency; HR_DP_: heart rate deflection point; KF_DP_: kick frequency at heart rate deflection point; K_TOTAL_: total number of kicks

Table 6 (and Additional file 2) provides correlations between sport-specific anaerobic (FSKT_10s_ and FSKT_mult_) and aerobic (PSTT) performance.

FSKT_TOTAL_ was correlated with aerobic power (KF_MAX_: ρ = 0.748 [95% CI: 0.387–0.910], *p* = 0.001, “*very large*”; K_TOTAL_: ρ = 0.759 [95% CI: 0.408–0.914], *p* = 0.001, “*very large*”; time to exhaustion: ρ = 0.730 [95% CI: 0.352–0.903], *p* = 0.001, “*very large*”) and capacity (HR_DP_: ρ = -0.606 [95% CI: -0.852 – -0.141], *p* = 0.013, “*large*”) indicators of the PSTT.

HR_MEAN_ and HR_PEAK_ of the FSKT_mult_ were correlated with HR_MAX_ (ρ = 0.656 [95% CI: 0.222–0.873], *p* = 0.006, “*large*”; ρ = 0.737 [95% CI: 0.366–0.906], *p* = 0.001, “*very large*”; respectively) and HR_DP_ (ρ = 0.623 [95% CI: 0.169–0.859], *p* = 0.010, “l*arge*”; ρ = 0.645 [95% CI: 0.205–0.869], *p* = 0.007, “l*arge*”; respectively) of the PSTT.

**Table 6:** Correlations between sport-specific anaerobic (FSKT_10s_ and FSKT_mult_) and aerobic (PSTT) performance in tested athletes (*n* = 16). Values are presented as spearman’s Rho coefficient [95% confidence intervals]

		Sport-Specific Aerobic Performance					
		HR_MAX_ (b.min^− 1^)	KF_MAX_ (k.min^− 1^)	HR_DP_ (b.min^− 1^)	KF_DP_ (k.min^− 1^)	K_TOTAL_ (n° kicks)	Time to exhaustion (s)
**Sport-Specific Anaerobic Performance**	FSKT_10s_ (n° kicks)	0.215 [-0.328–0.652]	0.588 [0.115–0.844]	-0.393 [-0.751–0.143]	0.191 [-0.351–0.637]	0.588 [0.115–0.844]	0.545 [0.052–0.825]
	FSKT_TOTAL_ (n° kicks)	-0.104 [-0.581–0.427]	0.748* [0.387–0.910]	-0.606* [-0.852 – -0.141]	0.555 [0.066–0.829]	0.759* [0.408–0.914]	0.730* [0.352–0.903]
	KDI (%)	0.402 [-0.133–0.756]	0.034 [-0.482–0.533]	-0.049 [-0.543–0.470]	-0.425 [-0.767–0.105]	0.001 [-0.507–0.509]	-0.029 [-0.529–0.486]
	HR_MEAN_ (b.min^− 1^)	0.656* [0.222–0.873]	-0.131 [-0.599–0.404]	0.623* [0.169–0.859]	-0.278 [-0.688–0.268]	-0.124 [-0.594–0.410]	-0.090 [-0.572–0.438]
	HR_PEAK_ (b.min^− 1^)	0.737* [0.366–0.906]	-0.144 [-0.608–0.392]	0.645* [0.205–0.869]	-0.336 [-0.721–0.208]	-0.136 [-0.602–0.400]	-0.119 [-0.591–0.414]

Notes: FSKT_10s_: total number of kicks in the 10 s Frequency Speed of Kick Test; FSKT_TOTAL_: total number of kicks in the 5 sets of the Multiple Frequency Speed of Kick Test; KDI: kick decrement index; HR_MEAN_: mean heart rate; HR_PEAK_: peak heart rate; HR_MAX_: maximal heart rate; KF_MAX_: maximal kick frequency; HR_DP_: heart rate deflection point; KF_DP_: kick frequency at heart rate deflection point; K_TOTAL_: total number of kicks. * = statistical significance following the Benjamini-Hochberg [40] correction procedure (*p* ≤ 0.013)

Table 7 presents correlations and comparisons of time-motion indexes between the tested athletes and their opponents during international matches.

Time-motion indexes were correlated (ρ = 0.756–1.000 [95% CI: 0.402–1.000], *p* < 0.001, from “*very large*” to “*perfect*”) and the activity profile did not differ (*p* > 0.05, “*small*”) between tested athletes and their opponents.

**Table 7:** Correlation coefficients (Spearman’s Rho) and comparisons (Mann-Whitney *U* test) of time-motion indexes between the tested athletes and their opponents during international matches. Values are presented as median (1st quartile–3rd quartile) [minimum–maximum]

Time-motion indexes	Total (*n* = 32)			Tested athletes (*n* = 16)			Opponents (*n* = 16)						
	Median	1st Q–3rd Q	Min–Max	Median	1st Q–3rd Q	Min–Max	Median	1st Q–3rd Q	Min–Max	ρ [95% CI]	U	*p*-value	*r*-ES
AT_AVG_ (s)	2.3	1.9–2.7	1.6–3.9	2.3	1.9–2.8	1.6–3.8	2.3	1.9–2.7	1.8–3.9	0.782* [0.456–0.923]	126.50	0.955	0.01
ST_AVG_ (s)	2.8	2.5–3.4	1.5–6.1	2.8	2.7–3.4	1.5–6.1	2.8	2.4–3.4	1.6–4.3	0.756* [0.402–0.913]	114.00	0.598	0.09
PT_AVG_ (s)	8.9	3.9–11.6	2.7–40.1										
AT_AVG_/ST_AVG_ ratio	0.75	0.57–1.23	0.35–2.17	0.74	0.57–1.04	0.35–2.17	0.76	0.59–1.32	0.42–1.73	0.835* [0.568–0.943]	114.50	0.611	0.09
(AT_AVG_ + ST_AVG_)/PT_AVG_ ratio	0.60	0.43–1.21	0.14–2.76	0.64	0.43–1.28	0.14–2.76	0.57	0.40–1.19	0.14–2.14	0.986* [0.958–0.995]	123.50	0.865	0.03
AT_SUM_ (s)	82.8	70.4–116.1	55.8–191.9	83.0	67.0–114.9	55.8–191.9	82.8	74.7–118.1	64.1–179.2	0.879* [0.672–0.959]	110.00	0.498	0.12
ST_SUM_ (s)	106.5	80.3–142.0	35.2–234.3	114.9	80.3–138.4	41.1–223.7	104.5	71.3–142.4	35.2–234.3	0.956* [0.871–0.985]	123.00	0.851	0.03
PT_SUM_ (s)	114.2	58.9–151.1	33.9–802.9										
AT_SUM_ (%)	43.6	36.6–56.9	26.7–70.4	42.8	36.6–53.4	26.7–70.4	43.6	37.1–62.0	29.8–66.9	0.874* [0.657–0.957]	114.00	0.598	0.09
ST_SUM_ (%)	56.4	43.1–63.4	29.6–73.3	57.2	46.6–63.4	29.6–73.3	56.4	38.1–62.9	33.1–70.2	0.874* [0.657–0.957]	114.00	0.598	0.09
AT_SUM_/ST_SUM_ ratio	0.77	0.58–1.33	0.36–2.37	0.75	0.58–1.15	0.36–2.37	0.78	0.59–1.64	0.43–2.02	0.870* [0.648–0.956]	115.00	0.624	0.09
(AT_SUM_ + ST_SUM_)/PT_SUM_ ratio	2.21	1.01–3.27	0.23–5.36	2.20	1.01–3.27	0.23–5.36	2.21	1.01–3.29	0.23–5.35	1.000* [1.000–1.000]	128.00	1.000	0.0001

Notes: AT_AVG_: average attack time; ST_AVG_: average skipping time; PT_AVG_: average pause time; AT_SUM_: sum of attack time; ST_SUM_: sum of skipping time; PT_SUM_: sum of pause time; ES: effect size. * = *p* < 0.001

Tables 8 and 9 (and Additional files 3 and 4) present correlations between sport-specific performance (FSKT_10s_, FSKT_mult_, and PSTT) and time-motion indexes.

No significant relationship emerged between FSKT performances and time-motion indexes (ρ = -0.442–0.396 [95% CI: -0.776–0.753, *p* > 0.05, “*moderate*”), as well as between PSTT indicators and time-motion indexes (ρ = -0.462–0.462 [95% CI: -0.786–0.786], *p* > 0.05, “*moderate*”).

**Table 8:** Correlations between sport-specific anaerobic (FSKT_10s_ and FSKT_mult_) performance and time-motion indexes during international matches in tested athletes (*n* = 16). Values are presented as spearman’s Rho coefficient [95% confidence intervals]

		Sport-Specific Anaerobic Performance				
		FSKT_10s_ (n° kicks)	FSKT_TOTAL_ (n° kicks)	KDI (%)	HR_MEAN_ (b.min^− 1^)	HR_PEAK_ (b.min^− 1^)
**Time-Motion Indexes**	AT_AVG_ (s)	-0.057 [-0.549–0.464]	-0.056 [-0.548–0.465]	-0.150 [-0.611–0.387]	-0.094 [-0.574–0.435]	-0.099 [-0.578–0.431]
	ST_AVG_ (s)	-0.148 [-0.610–0.389]	-0.155 [-0.615–0.383]	0.167 [-0.372–0.622]	-0.095 [-0.575–0.434]	-0.162 [-0.619–0.377]
	PT_AVG_ (s)	0.396 [-0.139–0.753]	0.298 [-0.247–0.700]	0.214 [-0.329–0.651]	0.271 [-0.275–0.685]	0.258 [-0.288–0.677]
	AT_AVG_/ST_AVG_ ratio	0.120 [-0.413–0.592]	0.112 [-0.419–0.587]	-0.109 [-0.584–0.422]	-0.033 [-0.532–0.483]	0.004 [-0.504–0.511]
	(AT_AVG_ + ST_AVG_)/PT_AVG_ ratio	-0.442 [-0.776–0.084]	-0.365 [-0.736–0.175]	-0.214 [-0.651–0.329]	-0.275 [-0.687–0.270]	-0.271 [-0.685–0.274]
	AT_SUM_ (%)	0.141 [-0.395–0.606]	0.151 [-0.386–0.612]	-0.106 [-0.582–0.425]	-0.042 [-0.538–0.476]	-0.015 [-0.519–0.497]
	ST_SUM_ (%)	-0.141 [-0.606–0.395]	-0.151 [-0.612–0.386]	0.106 [-0.425–0.582]	0.042 [-0.476–0.538]	0.015 [-0.497–0.519]
	AT_SUM_/ST_SUM_ ratio	0.141 [-0.395–0.606]	0.151 [-0.386–0.612]	-0.106 [-0.582–0.425]	-0.042 [-0.538–0.476]	-0.015 [-0.519–0.497]
	(AT_SUM_ + ST_SUM_)/PT_SUM_ ratio	-0.264 [-0.681–0.281]	-0.227 [-0.659–0.317]	-0.088 [-0.570–0.440]	-0.070 [-0.558–0.454]	0.025 [-0.489–0.526]

Notes: AT_AVG_: average attack time; ST_AVG_: average skipping time; PT_AVG_: average pause time; AT_SUM_: sum of attack time; ST_SUM_: sum of skipping time; PT_SUM_: sum of pause time; FSKT_10s_: total number of kicks in the 10 s Frequency Speed of Kick Test; FSKT_TOTAL_: total number of kicks in the 5 sets of the Multiple Frequency Speed of Kick Test; KDI: kick decrement index; HR_MEAN_: mean heart rate; HR_PEAK_: peak heart rate

**Table 9:** Correlations between sport-specific aerobic (PSTT) performance and time-motion indexes during international matches in tested athletes (*n* = 16). Values are presented as spearman’s Rho coefficient [95% confidence intervals]

		Sport-Specific Aerobic Performance					
		HR_MAX_ (b.min^− 1^)	KF_MAX_ (k.min^− 1^)	HR_DP_ (b.min^− 1^)	KF_DP_ (k.min^− 1^)	K_TOTAL_ (n° kicks)	Time to exhaustion (s)
**Time-Motion Indexes**	AT_AVG_ (s)	-0.053 [-0.546–0.467]	-0.226 [-0.658–0.318]	-0.155 [-0.614–0.383]	0.305 [-0.240–0.704]	-0.212 [-0.650– 0.332]	-0.228 [-0.660–0.316]
	ST_AVG_ (s)	-0.163 [-0.620–0.375]	-0.063 [-0.553–0.460]	-0.119 [-0.591–0.414]	-0.268 [-0.683–0.278]	-0.102 [-0.580–0.428]	-0.081 [-0.566–0.445]
	PT_AVG_ (s)	0.035 [-0.481–0.534]	0.037 [-0.480–0.535]	0.032 [-0.483–0.531]	0.223 [-0.321–0.656]	-0.006 [-0.512–0.503]	-0.031 [-0.530–0.484]
	AT_AVG_/ST_AVG_ ratio	0.075 [-0.450–0.562]	-0.060 [-0.551–0.462]	-0.139 [-0.604–0.396]	0.424 [-0.107–0.767]	-0.034 [-0.532–0.482]	-0.063 [-0.553–0.459]
	(AT_AVG_ + ST_AVG_)/PT_AVG_ ratio	-0.027 [-0.527–0.488]	-0.058 [-0.549–0.464]	-0.015 [-0.519–0.497]	-0.237 [-0.665–0.308]	-0.024 [-0.525–0.490]	0.003 [-0.506–0.510]
	AT_SUM_ (%)	0.052 [-0.468–0.545]	0.001 [-0.508–0.508]	-0.175 [-0.627–0.365]	0.462 [-0.06–0.786]	0.021 [-0.492–0.523]	0.001 [-0.508–0.508]
	ST_SUM_ (%)	-0.052 [-0.545–0.468]	0.001 [-0.508–0.508]	0.175 [-0.365–0.627]	-0.462 [-0.786–0.06]	-0.021 [-0.523–0.492]	0.001 [-0.508–0.508]
	AT_SUM_/ST_SUM_ ratio	0.052 [-0.468–0.545]	0.001 [-0.508–0.508]	-0.175 [-0.627–0.365]	0.462 [-0.06–0.786]	0.021 [-0.492–0.523]	0.001 [-0.508–0.508]
	(AT_SUM_ + ST_SUM_)/PT_SUM_ ratio	0.123 [-0.411–0.594]	-0.155 [-0.615–0.383]	0.091 [-0.437–0.573]	-0.295 [-0.698–0.250]	-0.121 [-0.592–0.412]	-0.116 [-0.589–0.416]

Notes: AT_AVG_: average attack time; ST_AVG_: average skipping time; PT_AVG_: average pause time; AT_SUM_: sum of attack time; ST_SUM_: sum of skipping time; PT_SUM_: sum of pause time; HR_MAX_: maximal heart rate; KF_MAX_: maximal kick frequency; HR_DP_: heart rate deflection point; KF_DP_: kick frequency at heart rate deflection point; K_TOTAL_: total number of kicks

## Discussion

The first aim of this study was to investigate the relationship between taekwondo sport-specific anaerobic and aerobic performance, using the FSKT to assess anaerobic power (FSKT_10s_) and capacity (FSKT_mult_), and the PSTT to determine aerobic capacity and power indicators. The first hypothesis was confirmed as significant and very large or large relationships between sport-specific anaerobic and aerobic performance emerged. Specifically, FSKT_TOTAL_ (i.e., the total number of kicks) was positively correlated with KF_MAX_ (i.e., the highest frequency of kicks reached in the last stage of the test), K_TOTAL_ (i.e., total number of kicks), and time to exhaustion of the PSTT. On the other hand, FSKT_TOTAL_ was negatively correlated with HR_DP_ (i.e., heart rate deflection point) of the PSTT. HR_MEAN_ and HR_PEAK_ of the FSKT_mult_ were positively correlated with HR_MAX_ and HR_DP_ of the PSTT. The second aim was to evaluate the validity of the above sport-specific tests in predicting the time-motion profile in international taekwondo matches. The second hypothesis was confirmed as no significant relationships between anaerobic and aerobic physical fitness variables and time-motion indexes of the match were found. In this sense, all time-motion indexes were positively and largely correlated and the activity profile did not differ significantly between the tested athletes and their opponents.

In the present study, the relationship between the two tests was examined by including the performance of both FSKT versions (FSKT_10s_ and FSKT_mult_) and all PSTT (submaximal and maximal) indicators in the investigation. The study of the relationships between different fitness components (e.g., anaerobic, aerobic, agility) is common in combat sports (such as judo and boxing), although these investigations generally involve both general and sport-specific tests [42–44]. In taekwondo, Albuquerque et al. [11] studied the relationship between FSKT_mult_ performance and some aerobic power indicators of the PSTT. A positive and significant relationship (*r* = 0.83) between FSKT_TOTAL_ and time to exhaustion of the PSTT emerged [11]. Current findings confirm this finding as FSKT_TOTAL_ was largely correlated with time to exhaustion of the PSTT and other maximal kick indicators (i.e., KF_MAX_ and K_TOTAL_). The studies that estimated the energy systems’ contributions during simulated matches widely recognized the central role of aerobic power in maintaining high-intensity, as it contributes to PCr resynthesis during low-intensity periods, pauses, and intervals between match rounds [3–6]. In this context, the pattern of correlations emerged supports the assumption that high aerobic power optimizes FSKT_TOTAL_ by allowing faster PCr resynthesis, during passive ten-second recoveries between high-intensity efforts [11], although no study to date has estimated the contributions of energy systems during the FSKT_mult_. In parallel, high anaerobic capacity (estimated by FSKT_TOTAL_) could result in better neuromuscular efficiency and tolerance to further increase of KF in the PSTT after maximal oxygen consumption (VO_2_MAX) has been reached [45]. In contrast, FSKT_10s_ was not correlated with any maximal kick indicators of the PSTT. In this sense, the performance achieved in a single all-out set of ten seconds is determined in particular by neuromuscular factors (e.g., high force production in small time intervals and recruitment of fast twitch muscle fibers) that go beyond aerobic fitness.

No significant correlation was found between FSKT_TOTAL_ and HR_MAX_ of the PSTT, both in the present study and previously [11]. Albuquerque et al. [11] hypothesized that the FSKT_mult_ is too short to allow for athletes to reach their HR_MAX_ and that the latter, derived from a continuous and progressive test, is unlikely to correlate with performance of a high-intensity intermittent test. Although current data confirm the inability to reach HR_MAX_ during the FSKT_mult_ (HR_PEAK_: ~96% of HR_MAX_ during the PSTT), it is important to highlight that HR_MEAN_ and HR_PEAK_ of the FSKT_mult_ were largely correlated with HR_MAX_ and HR_DP_ of the PSTT, as well as FSKT_TOTAL_ with HR_DP_ (i.e., an aerobic capacity indicator of the PSTT). In this regard, the lowest HR at the anaerobic threshold could indicate aerobic capacity as another key factor in optimizing the ability to repeat high-intensity efforts in the FSKT_mult_. As high lactate concentrations during the test (12.1–15.3 mmol∙L^− 1^) [8] and match (6.7–14.0 mmol∙L^− 1^) [1, 2] were reported, it is likely that these values were accompanied by a decrease in pH, and a high aerobic capacity would facilitate the recovery process between matches on competition day [7]. Therefore, the specific absence of a relationship between FSKT_TOTAL_ and HR_MAX_ of the PSTT might rather be related to the documented factors influencing this maximal indicator [46, 47].

In line with Albuquerque et a [11]. , KDI (i.e., kick decrement index) was not significantly correlated with any indicators of the PSTT. The absolute test-retest reliability findings (CV% >20) of this index corroborate the idea that it may not be adequate for identifying the benefits of aerobic power in the FSKT_mult_, as this variable considers all test sets and the possible variations that may occur in each [8, 11]. These previous absolute test-retest reliability findings highlight the importance of establishing the various aspects of validity, reliability, and sensitivity of sport-specific tests in parallel, as the lack of reliability of the primary test measurements could also negatively affect the validity of the analyses under study. Considering recent methodological advances in combat sports [12], the study of sensitivity (i.e., the degree to which the test detects small but meaningful changes in performance) and minimal detectable change (i.e., the estimate of the smallest change in score that can be detected objectively for a test) was incorporated into the analysis of intra-/inter-rater reliability, in line with recent studies that have demonstrated superior methodological approaches to reliability assessment [28, 29, 48]. The current findings showed that the FSKT_10s_ and FSKT_total_ have excellent relative and absolute intra-/inter-rater reliability and good sensitivity. In contrast, the KDI has acceptable absolute intra-/inter-rater reliability and marginal sensitivity.

The present study is the first that investigated the predictive criterion validity among sport-specific anaerobic and aerobic tests in taekwondo [7, 8]. For this purpose, the experimental design was based on official matches to improve experimental external validity, optimizing the applicability of the knowledge obtained to the official competitive environment. The findings revealed no significant relationship between anaerobic and aerobic fitness variables and time-motion indexes of the first match of the international competition day. In this context, several studies have shown the absence of a “pacing strategy” in relation to match outcome in international competitions [19, 20]. It has been hypothesized that the long and difficult selection processes minimize differences in performance ability among athletes in high-level competition [19]. Moreover, rule changes following the Tokyo 2020 Olympic Games (OG) have affected the duration of the match (with the introduction of the “best-of-three” system) to further increase fighting activity and consequently minimize the differences in the time structure of the athletes facing each other. Indeed, it was found that the athletes maintained a higher attack/skipping ratio (~ 1:1.3) than at the Tokyo 2020 OG (~ 1:1.5) [19]. In this regard, it should be clarified that in this Olympic competition, the winner was decided by the sum of points in the three rounds of the match (i.e., using the classic match structure), as well as the athletes underwent a rigorous qualification process to obtain Olympic participation (e.g., ranking criteria and continental qualification systems) [19], unlike those in the present study for the participation in the international competitions investigated (i.e., G-1/E-1 or E-2 competitions) [27]. Furthermore, all time-motion indexes were largely correlated and they did not differ significantly between the tested athletes and their opponents. Thus, the specific rhythm of the first official combat of the day could explain the inability to predict time and frequency spent in the different kinds of actions performed from the anaerobic and aerobic physical fitness variables. However, it is important to highlight that only the first match was analyzed, since each athlete performed a specific number of matches (based on the athletes in his/her weight category and/or number of matches won). In this sense, the conditioning level could have a different impact on the time structure of the match, when the athlete is required to perform 4–5 times on the day of competition, although the technical-tactical aspects could continue to shape the time and frequency spent in high- and low-intensity actions.

### Limitations and future directions

Although this study deals with important aspects related to sport-specific assessment in taekwondo, it has methodological limitations that warrant discussion.

- The sample includes a limited number of national/international athletes from the same club, aged between 15 and 23. These athletes’ characteristics limit the generalizability of the results to athletes of other competitive levels (e.g., state/regional), as well as to age categories such as cadets (i.e., 12–14 years old) and seniors (considering that the maximum age limit for this category is 35 years old). In this regard, the variation in athletes in terms of age category (i.e., junior and senior) could generate greater variation in performance and physiological test responses (attributable to potential biological maturity factors) and in time-motion indexes. These aspects emphasize the importance of analyzing their relationships independently as well.
- Athletes participated in an official international competition within 1–3 weeks after the testing sessions. It is important to highlight that, during this period, athletes continued to train regularly potentially modifying their conditioning status. In this context, the FSKT_TOTAL_ and KF_MAX_ performed by athletes are within the range of documented performances in national/international level athletes (56–125 kicks and 33–64 k.stage^-1^, respectively) [7, 8]. In this sense, a period of 1–3 weeks may have influenced the performance values of the tests.
- The PSTT has not been validated using electronic body protectors in order to positively impact the cost of the assessment [7]. Therefore, the use of traditional body protectors expands the evaluator’s task not only to checking the KF, but also the technical and power standard of each kick. Therefore, it is noteworthy that it is not possible to determine the intra-/inter-rater reliability of the PSTT without the use of electronic body protectors. In the present study, the problem was addressed through experimental sessions conducted by a researcher with ≥ 20 years of taekwondo experience and black belt.
- The use of sport-specific interval tests for endurance assessment (i.e., Interval Taekwondo-Specific Test, Interval Taekwondo-Specific Cardiopulmonary Test) [7], instead of continuous mode protocols (i.e., PSTT), could better reflect the physical and physiological demands of the sport by increasing the validity of the relationships between the aerobic and anaerobic components. Although these methodological alternatives demonstrate research efforts to improve the ecological validity of assessment, a recent review highlighted the need to confirm the validity of the fundamental aerobic variables of these tests [7]. On the contrary, indicators of aerobic capacity and power of the PSTT have been more studied and used to prescribe training [7].
- Time and frequency of different types of action recorded with time-motion analysis provide no direct information about the intensity applied, highlighting the need to also include other physiological (e.g., HR, blood lactate) and perceptual measurements (e.g., Borg 6–20 RPE scale) in the study of the predictive criterion validity of sport-specific tests. These additional measurements would also help explain the energy dynamics that support the maintenance of the high attack/skipping ratio.

### Practical applications

Despite the inability to predict the activity profile of the first official combat of the day from physical fitness variables, the time-motion indexes and the sport-specific tests’ performance can be used for prescribing highly specific and individualized trainings. Their applicability is supported by a simple and low-cost approach, aimed at satisfying the dynamics of interaction between the energy metabolisms for the optimal maintenance of typical combat intermittency [19, 49, 50].

- Sport-specific high-intensity interval training (HIIT) (i.e., involving kicks and punches), structured based on each athlete’s time-motion indexes, should help improve anaerobic capacity and consequently develop high aerobic power, which is useful for rapid PCr resynthesis during low-intensity periods and pauses [19].
- Sport-specific interval training (HIT), prescribed based on submaximal indicators derived from the PSTT (i.e., HR_DP_ and KF_DP_), should help improve aerobic capacity, thus retarding the glycolytic activity during match and supporting the pH recovery process between repeated matches. Recent studies have supported the validity of these HR-based indicators (i.e., HR_DP_ and KF_DP_) and tested the use of the *ITStriker* app in “training mode” to reproduce each athlete’s KF_DP_ during training [50, 51].
- Franchini et al. [49] suggested that the supramaximal performance of the FSKT_10s_ and the submaximal and maximal indicators of the PSTT (i.e., KF_DP_ and KF_MAX_, respectively) could be used to prescribe HIIT with short and long intervals (according to the classification proposed by Buchheit and Laursen [52]), adapting the concept of anaerobic speed/power reserve to the taekwondo mode.

## Conclusions

In conclusion, the pattern of correlations emerged between sport-specific anaerobic (FSKT) and aerobic (PSTT) performance suggests that the dynamics of interaction between anaerobic and aerobic metabolism are crucial for maintaining the short and intermittent kicking actions. In this sense, the future study of the energy systems’ contributions during the FSKT_mult_ will help to estimate the specific demands. The technical-tactical dynamics between the two interacting opponents, as a result of the most recent rule changes, give a specific rhythm to the time structure of the first international match of the competition day that justifies the inability to predict high- and low-intensity actions from the anaerobic and aerobic fitness variables. However, the athletes’ conditioning level would play a key role in supporting the high attack/skipping ratio (~ 1:1.3) of this specific match. Overall, physical fitness and technical-tactical aspects can be considered fundamental components for guaranteeing performance.

## Supplementary Information

Below is the link to the electronic supplementary material.

Supplementary Material 1

## Data Availability

The datasets used and/or analyzed during the current study are available from the corresponding author on reasonable request.

## Abbreviations

FSKT: Frequency Speed of Kick Test
PSTT: Progressive Specific Taekwondo Test
FSKT_10s_: 10 s Frequency Speed of Kick Test
FSKT_mult_: Multiple Frequency Speed of Kick Test
WT: World Taekwondo
FSKT_TOTAL_: Total number of kicks
KDI: Kick decrement index
HR_MEAN_: Mean heart rate
HR_PEAK_: Peak heart rate
%HR_MAX_: Percentages of the actual maximal heart rate in the PSTT
ICC: Intraclass correlation coefficient
CV: Coefficient of variation
SEM: Standard error of measurement
SWC: Smallest worthwhile change
MDC_95_: Minimal detectable change at 95% confidence interval
HR_MAX_: Maximal heart rate
KF_MAX_: Maximal kick frequency
HR_DP_: Heart rate deflection point
KF_DP_: Kick frequency at the HR_DP_
K_TOTAL_: Total number of kicks
AT: Attack time
AN: Number of attack time
ST: Skipping time
PT: Pause time
VO_2_MAX: Maximal oxygen consumption
OG: Olympic Games
HIIT: High-intensity interval training
