# Supplementary material for: Relationships between sport-specific tests and their validity in predicting the time-motion profile in international taekwondo matches
Source: BMC Sports Sci Med Rehabil. 2025 Nov 10;17:323. doi: 10.1186/s13102-025-01371-4 (PMC12599103; doi:10.1186/s13102-025-01371-4)
Supplement: Supplementary file 1 — Supplementary Material 1 [file 13102_2025_1371_MOESM1_ESM.pdf]

Equation (1):

$$KDI\ (\%) = \left[1 - \frac{\text{FSKT1} + \text{FSKT2} + \text{FSKT3} + \text{FSKT4} + \text{FSKT5}}{\text{best FSKT} \times \text{number of sets}}\right] \times 100$$

Additional file 1. Kick decrement index (KDI) equation.

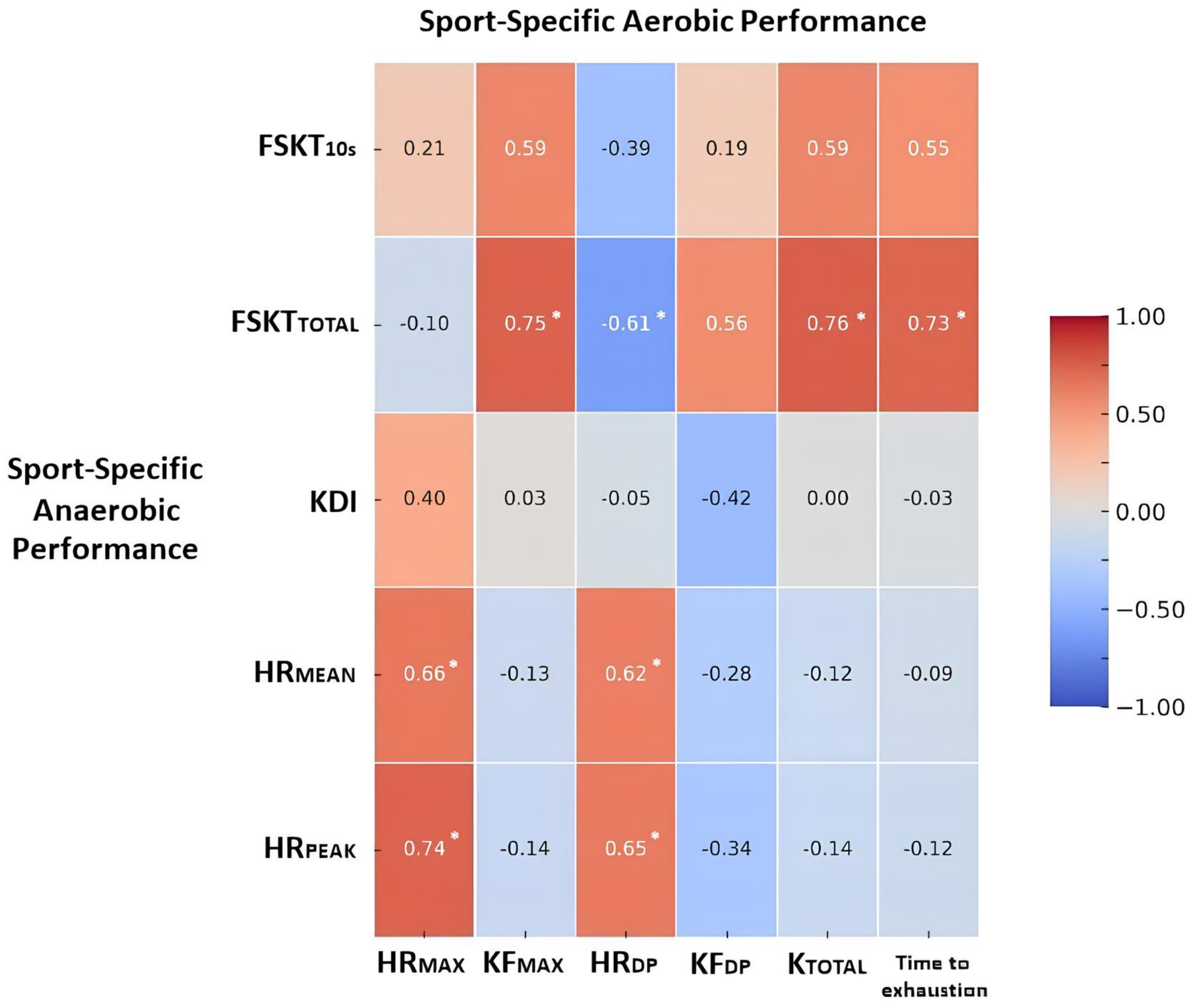

Additional file 2. Correlations between sport-specific anaerobic ($FSKT_{10s}$ and $FSKT_{mult}$) and aerobic (PSTT) performance in tested athletes (n = 16). Values are presented as Spearman's Rho coefficient.

Notes: $FSKT_{10s}$: total number of kicks in the 10 seconds Frequency Speed of Kick Test; $FSKT_{TOTAL}$: total number of kicks in the 5 sets of the Multiple Frequency Speed of Kick Test; KDI: kick decrement index; HR_{MEAN}: mean heart rate; HR_{PEAK}: peak heart rate; HR_{MAX}: maximal heart rate; KF_{MAX}: maximal kick frequency; HR_{DP}: heart rate deflection point; KF_{DP}: kick frequency at heart rate deflection point; K_{TOTAL}: total number of kicks. * = statistical significance following the Benjamini-Hochberg correction procedure ($p \leq 0.013$).

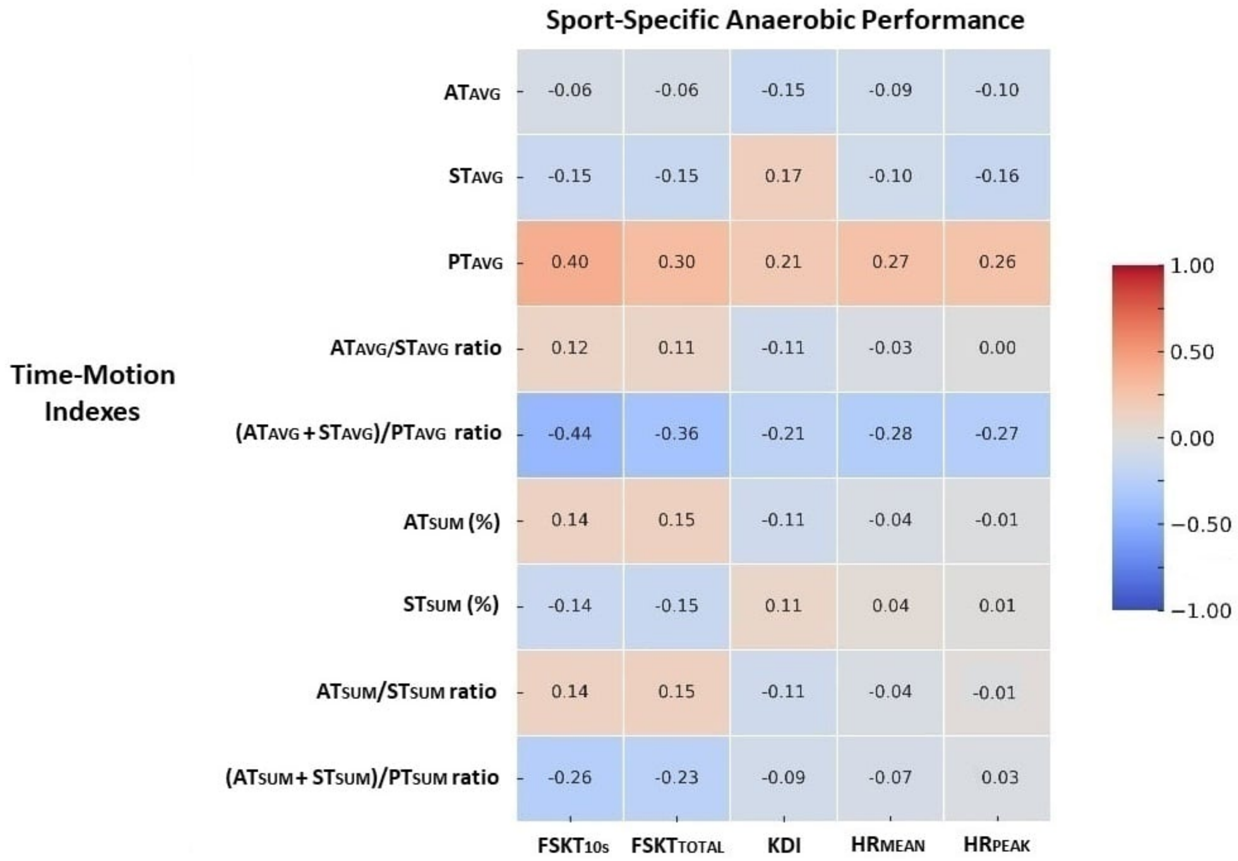

Additional file 3. Correlations between sport-specific anaerobic ($FSKT_{10s}$ and $FSKT_{mult}$) performance and time-motion indexes during international matches in tested athletes (*n* = 16). Values are presented as Spearman's Rho coefficient.

Notes: AT_{AVG}: average attack time; ST_{AVG}: average skipping time; PT_{AVG}: average pause time; AT_{SUM}: sum of attack time; ST_{SUM}: sum of skipping time; PT_{SUM}: sum of pause time; $FSKT_{10s}$: total number of kicks in the 10 seconds Frequency Speed of Kick Test; $FSKT_{TOTAL}$: total number of kicks in the 5 sets of the Multiple Frequency Speed of Kick Test; KDI: kick decrement index; HR_{MEAN}: mean heart rate; HR_{PEAK}: peak heart rate.

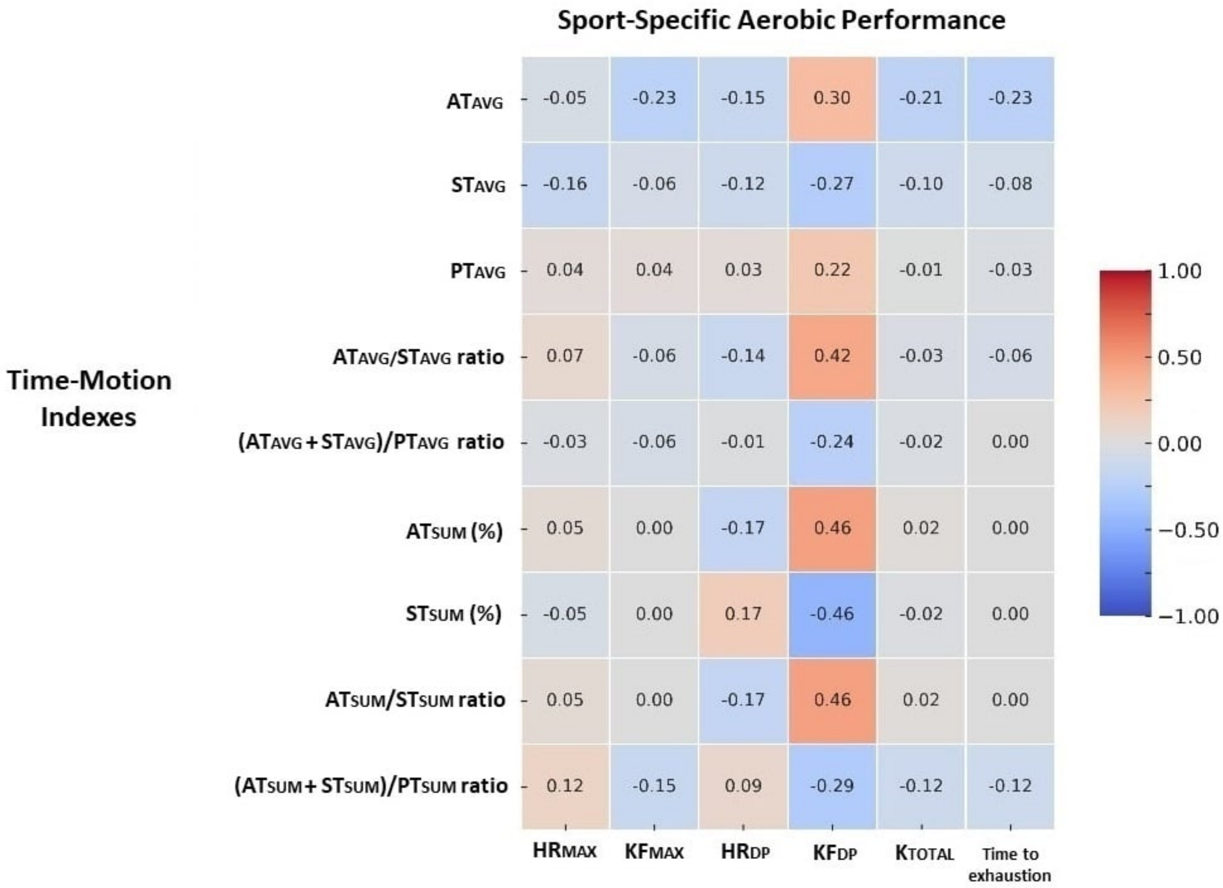

Additional file 4. Correlations between sport-specific aerobic (PSTT) performance and time-motion indexes during international matches in tested athletes (*n* = 16). Values are presented as Spearman's Rho coefficient.

Notes: AT_{AVG}: average attack time; ST_{AVG}: average skipping time; PT_{AVG}: average pause time; AT_{SUM}: sum of attack time; ST_{SUM}: sum of skipping time; PT_{SUM}: sum of pause time; HR_{MAX}: maximal heart rate; KF_{MAX}: maximal kick frequency; HR_{DP}: heart rate deflection point; KF_{DP}: kick frequency at heart rate deflection point; K_{TOTAL}: total number of kicks.
